# Thermal constraints on exercise and metabolic performance do not explain the use of dormancy as an overwintering strategy in the cunner (*Tautogolabrus adspersus*)

**DOI:** 10.1242/jeb.246741

**Published:** 2024-01-11

**Authors:** Lauren E. Rowsey, Connor Reeve, Tyler Savoy, Ben Speers-Roesch

**Affiliations:** Department of Biological Sciences, University of New Brunswick Saint John, 100 Tucker Park Road, Saint John, NB E2L 4L5, Canada

**Keywords:** Metabolic rate, Burst swimming, Aerobic scope, Acclimation, Hibernation, Temperature, Winter, Fish

## Abstract

Winter cold slows ectotherm physiology, potentially constraining activities and ecological opportunities at poleward latitudes. Yet, many fishes are winter-active, facilitated by thermal compensation that improves cold performance. Conversely, winter-dormant fishes (e.g. cunner, *Tautogolabrus adspersus*) become inactive and non-feeding overwinter. Why are certain fishes winter-dormant? We hypothesized that winter dormancy is an adaptive behavioural response arising in poleward species that tolerate severe, uncompensated constraints of cold on their physiological performance. We predicted that below their dormancy threshold of 7­–8°C, exercise and metabolic performance of cunner are greatly decreased, even after acclimation (i.e. shows above-normal, uncompensated thermal sensitivity, *Q*_10_>1–3). We measured multiple key performance metrics (e.g. C-start maximum velocity, chase swimming speed, aerobic scope) in cunner after acute exposure to 26–2°C (3°C intervals using 14°C-acclimated fish) or acclimation (5–8 weeks) to 14–2°C (3°C intervals bracketing the dormancy threshold). Performance declined with cooling, and the acute *Q*_10_ of all six performance rate metrics was significantly greater below the dormancy threshold temperature (*Q*_10,acute8–2°C_=1.5–4.9, mean=3.3) than above (*Q*_10,acute14–8°C_=1.1–1.9, mean=1.5), inferring a cold constraint. However, 2°C acclimation (temporally more relevant to seasonal cooling) improved performance, abolishing the acute constraint (*Q*_10,acclimated8–2°C_=1.4–3.0, mean=2.0; also cf. *Q*_10,acclimated14–8°C_=1.2–2.9, mean=1.7). Thus, dormant cunner show partial cold-compensation of exercise and metabolic performance, similar to winter-active species. However, responsiveness to C-start stimuli was greatly cold-constrained even following acclimation, suggesting dormancy involves sensory limitation. Thermal constraints on metabolic and exercise physiology are not significant drivers of winter dormancy in cunner. In fact, compensatory plasticity at frigid temperatures is retained even in a dormant fish.

## Teaser

**Summary:** Thermal constraints on metabolic and exercise physiology are not significant drivers of winter dormancy in cunner. In fact, compensatory plasticity at frigid temperatures is conserved, even in dormant fish.

## INTRODUCTION

The ability to cope with cold, and food-poor, winters is a key to persistence of animal populations at poleward latitudes (Marchand, 2014; Feary et al., 2014; Stuart-Smith et al., 2017). For ectotherms, frigid winter temperatures, in particular, can greatly constrain performance, activity and fitness (Ultsch, 1989; Shuter et al., 2012; Huey, 2010). Three main overwintering strategies have evolved to cope with winter and maintain a poleward range: (1) seasonal movement to warmer areas (cold avoidance), (2) maintenance of activity by compensation of physiology (i.e. improved cold performance) via cold acclimation and/or adaptation, and (3) an inactive, energy-saving state of dormancy (Marchand, 2014; Shuter et al., 2012; White et al., 2012). Winter dormancy in ectotherms is an inactive, sheltering, low metabolic rate and non-feeding state, generally analogous to mammalian hibernation (Gregory, 1982; Reeve et al., 2022; Ultsch, 1989). Among fishes, winter dormancy occurs in numerous temperate species but contrasts with the more common, and better studied, strategy of sustained winter activity supported by physiological thermal compensation (Amundsen and Knudsen, 2009; Block et al., 2020; Campbell et al., 2008; Crawshaw, 1984; Guderley, 1990; Reeve et al., 2022). Little is known about the driver(s) of winter dormancy in fishes or other vertebrate ectotherms – why do certain species use dormancy as a strategy to survive winter?

**List of symbols and abbreviations**

AAS: absolute aerobic scope
*A*_max_: maximum acceleration
BL: body length
CK: creatine kinase
CrP: creatine phosphate
CS: citrate synthase
EPOC: excess post-exercise oxygen consumption
LDH: lactate dehydrogenase
MMR: maximum metabolic rate
*Ṁ*_O_2__: oxygen consumption rate
*Q*_10_: thermal sensitivity quotient
SMR: standard metabolic rate
*T*_opt_: thermal optimum
*U*_chase_: annular chase swimming speed
*U*_crit_: maximum sustained swimming speed
*U*_max_: instantaneous maximum burst velocity
WDTT: winter dormancy threshold temperature

**Fig. 1.**
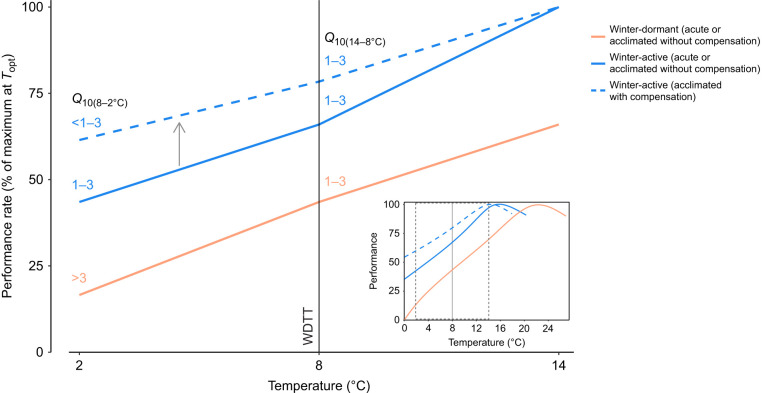
**Predictions of the thermal performance of fish species with differing overwintering strategies (winter-active versus -dormant) following our ‘cold performance limitation’ hypothesis (i.e. winter dormancy is an adaptive behavioural response arising in species that tolerate severe, uncompensated constraints of cold on physiological performance that would make winter activity perilous).** The solid lines represent the predicted performance of winter-dormant species (orange) or winter-active species (blue) during acute cooling of warm-acclimated (here, 14°C) fish towards a winter temperature (2°C) or following uncompensated acclimation to lowered temperatures; the associated *Q*_10_ values above each line are for the temperature intervals 8–2°C and 14–8°C [below and above the winter dormancy threshold temperature (WDTT); here, 8°C, similar to cunner (Reeve et al., 2022)]. Performance of the winter-dormant species is lower at any given temperature because we expect it to have a warm-shifted thermal performance curve (inset; where data shown in the main panel are outlined with dashed grey box), such that its performance at winter temperatures overlaps that at the constrained, extreme cold end of its thermal window. Thus, we predict the dormant species' performance to follow a typical, passive *Q*_10_ of 1–3 (here, modelled as *Q*_10_=2; Seebacher et al., 2015) with cooling above the WDTT (black line) versus a greater *Q*_10_ (specifically, *Q*_10_>3, suggesting a constraint on physiological performance; modelled as *Q*_10_=5 in main panel) as performance approaches zero with cooling below the WDTT. In contrast, we expect the winter-active species to have, at least, a consistent passive *Q*_10_ of 1–3 (here, 2) with cooling to 2°C. Acclimation of the winter-active species to cooler temperatures (e.g. 8°C, 2°C) may additionally result in partial thermal compensation that enhances performance (grey arrow) and consequently lowers thermal sensitivity (here, *Q*_10_=1.5) (dashed blue line), while the dormant species' performance would remain uncompensated following acclimation.

We propose the ‘cold performance limitation’ hypothesis (Fig. 1) whereby winter dormancy in fishes, and possibly other vertebrate ectotherms, is an adaptive behavioural response to severe, uncompensated constraints of cold on physiological performance that make it inefficient and/or risky to remain active in winter. In other words, winter dormancy is an alternative strategy to physiological compensation, allowing a species to tolerate its sluggish, inflexible performance in seasonal cold by pausing life activities and sheltering in an energy-savings mode. Essentially, winter dormancy could be a ‘behavioural filter’ (Garland and Losos, 1994) that compensates for poor cold performance. Dormancy theoretically could be advantageous by minimizing acclimation costs (Wilson and Franklin, 2002) and may be an acceptable trade-off for better performance at warm temperatures (Malusare et al., 2023). Indeed, we expect that dormant species, which tend to belong to ‘warm-adapted’ lineages (e.g. Labridae), have cold-constrained performance because they have warm-shifted performance curves, such that winter temperatures overlap the extreme cold end of their thermal window where performance nears zero (Fig. 1). The cold performance limitation hypothesis for winter dormancy, also alluded to by Shuter et al. (2012) and hinted at by reptile researchers for decades (Cowles, 1941; Gregory, 1982; Huey and Pianka, 1977; Huey and Stevenson, 1979; Huey et al., 2021), contrasts with (but is not exclusive of) the ‘energy availability’ hypothesis for mammalian hibernation whereby food scarcity drives dormancy (Humphries et al., 2003). Constraints of cold on performance can powerfully influence activity, habitat selection, predation risk and anti-predator behaviours among ectotherms (Bennett, 1990; Gunderson and Leal, 2016; Herrel et al., 2007; Huey, 1982; Huey and Stevenson, 1979; Kingsolver, 1983; Rand, 1964). However, the cold performance limitation hypothesis has not been investigated in ectotherms as an explanation for the widespread phenological event of winter dormancy.

Aerobic and exercise performance are ideal traits to investigate the cold performance limitation hypothesis because they are fitness-linked, temperature-dependent traits in ectotherms including fishes (Kieffer, 2010; Schulte, 2015). Sustained and burst swim performance, standard (SMR) and maximum metabolic rates (MMR), and aerobic scope (=MMR–SMR, the aerobic capacity for life activities such as swimming) all usually decrease with cooling below their optimal temperature (*T*_opt_) in fish species; however, winter-active species such as salmonids show well-maintained swimming and aerobic scope when exposed to low temperatures that may be further ameliorated by cold acclimation (Guderley, 1990; Hvas et al., 2017, 2018; Johnston and Temple, 2002; Porter and Gamperl, 2023; Schurmann and Steffensen, 1997; Tirsgaard et al., 2015). Although variation in the cold sensitivity of metabolic performance is known among fishes (Guderley, 1990), temperature–performance relationships have rarely been interpreted in the context of species-specific overwintering strategies and have not often been studied at winter low temperatures (<5°C).

Here, we tested the cold performance limitation hypothesis for winter dormancy in fishes. We predicted that large and uncompensated decreases in physiological performance would occur at temperatures below the winter dormancy threshold temperature (WDTT: the temperature below which there are disproportionately great decreases in activity and feeding as the fish transitions into dormancy; Reeve et al., 2022) (Fig. 1). We studied cunner (*Tautogolabrus adspersus*), a temperate wrasse with a striking winter dormancy involving inactivity and fasting below their population-specific WDTT of 5–8°C (Bradbury and Green, 1997; Reeve et al., 2022; Speers-Roesch et al., 2018). Focusing on exercise and metabolic performance, we completed two experiments to assess acute and acclimated thermal performance in cunner from Nova Scotia (WDTT=7–8°C; Reeve et al., 2022): (1) swim speed performance (C-start burst escape response and swim speed during exhaustive chase), and (2) whole-animal exhaustive exercise endurance (time to exhaustion from chase), whole-animal aerobic performance (MMR and aerobic scope), exercise recovery [excess post-exercise O_2_ consumption (EPOC) and its duration] and underlying muscle metabolic responses (aerobic and anaerobic enzyme activities, fuel metabolites). We used the thermal sensitivity quotient (*Q*_10_), which describes the temperature dependency of a biological rate (Clarke, 2017), to evaluate whether thermal constraints on performance drive winter dormancy. Thermal sensitivity of metabolism and locomotor performance in ectotherms is normally associated with *Q*_10_=1–3, whereas a higher *Q*_10_ suggests a constraint (Clarke, 2017; Jørgensen et al., 2022; Peck, 2016; Seebacher et al., 2015). We predicted that greater decreases in performance, and consequently higher *Q*_10_ (specifically, and conservatively, >3), would occur with cooling below the WDTT than above it (where conservatively, we expected *Q*_10_ <3). We measured exercise recovery to test the prediction that potential thermal constraints on aerobic performance would translate into disproportionately greater EPOC (i.e. a greater cost for recovery) and/or longer recovery time in the cold. Finally, by comparing performance levels and *Q*_10_ values of cunner acclimated to cooled temperatures with those of warm-acclimated (14°C) cunner acutely exposed to the same cooled temperatures, we evaluated whether compensation via plasticity (i.e. increased performance and reduced thermal sensitivity following acclimation) occurred below the WDTT; we expected to find no evidence of compensation (i.e. unchanged performance level and unchanged *Q*_10_ between acute and acclimated exposures) (Fig. 1). Acute and acclimated thermal responses are rarely compared within the same study, despite the value for revealing plasticity (Havird et al., 2020). By studying dormant species such as cunner, we can provide insight into the origins of divergent thermal responses to cold among fishes.

## MATERIALS AND METHODS

### Animals

Juvenile cunner [*Tautogolabrus adspersus* (Walbaum 1792)] were obtained from the Cooke Aquaculture captive breeding program at the Huntsman Marine Science Centre in St Andrews, New Brunswick, in September 2017 and June 2018 (F1 offspring of wild-caught parents from St Mary's Bay, Nova Scotia; see Table S1 for fish body masses and lengths for each experiment). Fish were transferred to 165 litre fiberglass circular tanks supplied with aerated, filtered seawater from a recirculating seawater system (14±0.5°C) at the University of New Brunswick Saint John. Fish were fed every second day to satiation with commercial pellets (Gemma Diamond 1.8 mm; Skretting, St Andrews, NB, Canada). The fish were kept on a winter photoperiod typical of Atlantic Canada (10 h:14 h light:dark), which included a simulated sunrise and sunset (30 min each) with sunrise/sunset lamps (WADEO, Brooklyn, NY, USA) to minimize disturbances from sudden light changes. This winter photoperiod also was used in all experiments. Cunner remain active and feed under a winter photoperiod, so long as the temperature is above their WDTT of 7–8°C (Reeve et al., 2022). Cunner were acclimated to these holding conditions for at least 6 weeks before experiments began.

### Overview of temperature exposures and experimental acclimation systems

We ran two experiments, outlined below, using a common temperature exposure protocol for measuring thermal performance which involved acutely challenging (3°C h^−1^) 14°C-acclimated cunner to a broad range of temperatures (26–2°C in 3°C increments, with a different group of 14°C-acclimated fish acutely exposed to each temperature) as well as acclimation (5–8 weeks, after a gradual 0.5°C day^−1^ cooling) of cunner to a range of temperatures relevant to the summer to winter transition (14–2°C in 3°C increments, with a different group of fish acclimated to each temperature) (see ‘Experiment 1’ for more information, and ‘Experiment 1’, ‘Experiment 2A’ and ‘Experiment 2B’ for experiment-specific details). The slower acclimation cooling rate of 0.5°C day^−1^ and up to 8 weeks of acclimation better approximates seasonal cooling while keeping acclimation duration logistically feasible (Huey and Buckley, 2022). Temperatures of 14°C and 2°C are common summer and winter ocean temperatures, respectively, encountered by cunner across their range. The upper and lower thermal limits of cunner are roughly 28°C (Kelly et al., 2014) and 0°C (Green and Farwell, 1971). Thus, our acute temperature exposure range (26–2°C) captures nearly the entire thermal tolerance range of cunner, and our lowest acute/acclimated temperature of 2°C falls at the extreme cold end of their thermal performance curve. A broader range of temperatures was used for acute exposures to characterize the acute thermal performance curve for each performance metric including *T*_opt_ and thermal sensitivity over a broader range of warm, active temperatures (see ‘Thermal sensitivity analysis’ section); the acclimation exposures, which are more space- and time-intensive, focused on the temperatures that equally bracket the WDTT (±6°C around the WDTT of approximately 8°C; Reeve et al., 2022). Although a fully factorial design involving acute exposures of each acclimation group to all other test temperatures provides the most robust assessment of thermal plasticity (Havird et al., 2020), this is logistically challenging with relatively large fish such as cunner. Thus, we used our simplified multi-temperature design involving comparisons of 14, 11, 8, 5 or 2°C-acclimated fish with 14°C-acclimated fish acutely exposed to the same acclimation temperatures; the difference in the measured variable between the acute and acclimated exposures at the same temperature inferred the extent of plasticity.

Cunner were transferred from holding tanks to dedicated experimental aquarium systems for temperature acclimation prior to physiological performance measurement. Each acclimation system corresponded to a specific acclimation temperature and consisted of a recirculating seawater system that supplied four glass aquaria per system (or six aquaria at 14°C to provide room for the additional fish required for acute temperature exposures). Each aquarium contained 60 litres of seawater and three PVC pipe shelters (each 10×2.5 cm, length×diameter). Fish were equally distributed among aquaria (14 fish per tank initially); later, for acute or acclimated performance measurements, fish were taken from randomly selected aquaria from the 14°C-acclimated group (for measurement of acutely exposed fish) or from each acclimation temperature (for measurement of acclimated fish). The seawater within each system was temperature controlled to ±0.5°C with a 1/3 HP Arctica chiller (JBJ Chillers, St Charles, MO, USA) and water quality was maintained via active biomedia, mechanical filtration, constant aeration and occasional seawater replacement (Arctica water chillers also were used for all experiments described below). All acclimation systems were housed in the same wet lab room. Fish were fed every second day to satiation on commercial pellets (but rarely ate below ∼8°C) and fasted for 48 h before measurements of performance. All experimental procedures were approved by the animal care committee of the University of New Brunswick in compliance with Canadian Council of Animal Care guidelines.

### Experiment 1: swim performance (**C**-start escape response and annular chase swimming speed)

Swim speed performance was assessed using two metrics: (1) instantaneous maximum burst velocity [*U*_max_; body lengths (BL) s^−1^], during the anaerobic C-start escape response (Domenici and Blake, 1997), and (2) annular chase swimming speed (*U*_chase_; BL s^−1^), which includes aerobic and, especially, anaerobic swimming and was achieved using a variation of the standardized chase method where fish were chased in laps around a circular raceway until exhaustion (Norin and Clark, 2016). We measured *U*_chase_, as opposed to *U*_crit_ (maximum sustained swimming speed), because it allows assessment of swim velocity performance in species that swim poorly in swim tunnels (Norin and Clark, 2016). During preliminary trials with cunner, we found it difficult to encourage them to swim continuously in a tunnel, similar to other temperate wrasse species (Yuen et al., 2019).

Different groups of cunner were acclimated to either 14, 11, 8, 5 or 2°C for 5–6 weeks (*n*=12 per temperature; see Table S1 for body masses and lengths), and another group of cunner was acclimated to 14°C and then acutely exposed to either 26, 23, 20, 17, 14 (as a sham control for the acute exposure procedure), 11, 8, 5 or 2°C (*n*=12 per temperature; see Table S1 for body masses and lengths). Each acclimation system (described above) was cooled at a rate of 0.5°C day^−1^ until the acclimation temperature was reached (excluding 14°C), with cooling start dates staggered so all acclimation groups reached their acclimation temperature on the same date. Acute trial fish were taken from the 14°C acclimation system and cooled or warmed to their test temperature at 3°C h^−1^ (over a fixed 4-h period, e.g. 8°C-exposed fish were held at 14°C for 2 h and then cooled over 2 h; 14°C-exposed fish were held at 14°C for 4 h, etc.) in a pre-test holding system adjacent to the test arenas (see below); the fish then remained at their test temperature for 1 h before the swim test (total of 5 h before the test). Acclimated fish were also placed in the same pre-test holding system for a 5-h period at their respective acclimation temperature prior to swim tests. The pre-test holding system consisted of a 75 litre glass aquarium (where fish were held) that drained into a sump (55×42×20 cm, diameter×height×water depth) containing a submersible pump that transferred aerated temperature-controlled seawater back to the glass aquarium.

Each fish went through two swimming speed tests beginning with the C-start test (to measure *U*_max_) followed by the annular chase test (to measure *U*_chase_). C-start trials were carried out in a large fibreglass tank (30×85 cm, depth×diameter) filled to 15 cm depth with aerated temperature-controlled seawater (the C-start arena). The seawater in the arena drained to an insulated sump and seawater was recirculated back to the arena via a chiller by a submersible sump pump. The pump had a bypass back to the sump to ensure water flow into the arena was low and seawater inflow to the arena was removed during the trial (i.e. static system during C-start attempts). A high-speed digital video camera (240 frames s^−1^, Olympus Tough TG-5; Olympus, Shinjuku, Tokyo, Japan) was mounted 115 cm above the setup by attaching a flexible tripod to a PVC pipe frame. A white corrugated flat plastic sheet marked with a line of known distance (3 cm; for distance calibration) was fitted to the bottom of the circular arena to provide the fish with good contrast. A PVC pipe (75×3.8 cm, length×diameter) was fastened vertically to the inside of the tank, into which a weighted Falcon tube (38 g) could be dropped into the tank water to provide a mechano-acoustic stimulus to startle the fish and elicit a C-start response. A 65 cm long string was tied to the Falcon tube to ensure only the tube tip entered the water and which allowed inconspicuous retrieval of the tube before the next trial. This entire system was enclosed by black plastic bags to minimize outside disturbances and eliminate glare on the water from overhead lights.

Prior to beginning the C-start test, individual fish were transferred via net immersed in seawater from the pre-experiment holding system to the C-start arena and allowed 30 min to adjust to their surroundings. Then, once the fish was within ∼40 cm (radius of tank) of the PVC pipe, video recording commenced followed by dropping of the weighted Falcon tube (a mechano-acoustic stimulus). An attempt was deemed successful if the fish elicited a C-start response and a total of three attempts using the weighted tube drop were completed (regardless of a successful C-start) with 5 min rest periods between each (Domenici and Blake, 1997; Marras et al., 2011). During preliminary tests at 2°C, some fish did not react to the tube drop during the initial three attempts; therefore, an additional two attempts were employed on all fish at all temperatures whereby 20 cm long forceps were used to pinch the fish's caudal fin quickly and gently. At all temperatures, this procedure elicited a C-start, though some fish would react and burst before contact; in either scenario, any identified C-starts were analyzed. Subsequent statistical analysis showed no significant effect of stimulus type on the C-start variables of *U*_max_ or maximum acceleration (*A*_max_) (*P*=0.184 and 0.655, respectively; ANOVA). Pre-simulation variables including distance to the arena wall, distance to the stimulus, and angle of the fish relative to the stimulus are provided in Table S2. Distance to stimulus or wall were not significantly different (*P*=0.152 and 0.159, respectively; ANOVA) between acute and acclimated treatments, and thus were unlikely to influence our evaluation of acclimation effects. Distance to stimulus varied significantly (*P*<0.05) across temperatures owing to the increased need to use forceps (which approached the fish more closely) to stimulate fish at the coldest temperatures. Distance to wall also varied significantly across temperatures, owing to higher values at the coldest temperature; however, successful C-starts occurred in the direction away from the wall, so it is unlikely to have impeded the performance. Furthermore, if any effect occurred, a greater distance to wall would likely result in higher *U*_max_ or *A*_max_, yet the coldest fish still showed the lowest burst performance; thus, at worst, we conservatively estimated the cold constraint. Finally, stimulus angle varied significantly between treatments as well as across temperatures, again because of increased forceps use at the coldest temperatures, especially in acutely cooled fish; forceps were directed at the tail (180 deg stimulus angle) so resulted in higher values for stimulus angle. Because forceps stimulation was necessary at colder temperatures, the effect of their use on pre-stimulation variables was unavoidable, but is unlikely to have had meaningful effects on our measurements of *U*_max_ and *A*_max_ for the reasons above and also because *U*_max_ or *A*_max_ were not affected by stimulus type and C-starts are stereotyped, neuromuscular reflexes once triggered.

Following the last C-start attempt, the fish was transferred via net in seawater to the adjacent annular swim arena and allowed a 30 min recovery period before measuring *U*_chase_. The annular swim arena consisted of a round bucket (55×42 cm, diameter×height) containing aerated, temperature-controlled seawater, with a smaller white bucket (24×21.5 cm, diameter×height; weighted with seawater) centered within it; this created a circular loop between the walls of the two buckets where the fish could swim (dimensions of the swimming zone: 15.5×176×20 cm, width×circumference×water depth). The submersible pump recirculating seawater to a chiller was removed following the recovery period so that during the test the fish could swim around the circular loop without obstacles. A manually operated PVC pipe (80×1.9 cm, length×diameter) was used to frequently make contact with the fish's caudal fin to elicit bursts and sustained swimming until the fish no longer responded to five contacts, which we defined as exhaustion. Each test was recorded using a digital video camera (Olympus Tough TG-5; Olympus, Shinjuku, Tokyo, Japan) mounted above using a tripod, and the videos were analyzed later for total laps swam over the time from start of chase to time to exhaustion.

The C-start *U*_max_ and *A*_max_ for each fish was measured from the high-speed videos of C-start responses using the automated tracking software Tracktor (Sridhar et al., 2019; https://github.com/vivekhsridhar/tracktor). The video analysis software Tracker (v5.1.5; https://physlets.org/tracker/) was used for videos where poor contrast made it impossible for Tracktor to detect the fish. Comparisons of the output from the same videos analyzed with both software confirmed that measured parameters were comparable in both cases. Pixel-to-distance calibrations were done in each software using the 3 cm line drawn on bottom of arena. To measure *U*_max_ and *A*_max_, a common time period was analyzed for each fish (75 ms) following initiation of the C-start; this corresponded to the average time for each fish between initiation of stage 1 of the C-start response until the completion of stage 2 (Domenici and Blake, 1997). In Tracker, we used point mass analysis with the center of the fish's head between the eyes set as the reference point. Measurement of *U*_max_ and *A*_max_ was carried out for each C-start obtained from the 5 attempts per fish, and the highest value of *U*_max_ and the associated *A*_max_ were recorded (i.e. both values were from the same attempt). *U*_max_ was measured in cm s^−1^ and standardized to the individual's body length (and reported as BL s^−1^) and *A*_max_ was measured in cm s^−2^ and reported as m s^−2^.

We also measured C-start responsiveness for each fish, which was calculated as the percentage of total attempts that elicited a C-start response to the weighted tube drop or approach of the forceps during the pinch attempt (i.e. prior to direct tactile contact). By only considering C-starts elicited prior to contact with the fish to be successful responses, our measure of responsiveness reflects visual and/or motion sensing rather than tactile responsiveness. In fact, all fish that were contacted with the forceps responded with a C-start, indicating the robustness of tactile stimuli in triggering a C-start regardless of temperature exposure.

Annular chase swimming speed (*U*_chase_) was defined as the total number of laps swam per unit time over the entire chase duration (i.e. until time of exhaustion) standardized to body length (BL s^−1^).

### Experiment 2A: exhaustive exercise endurance and aerobic performance

We used the standardized chase method followed by respirometry (Norin and Clark, 2016) to assess: (1) exhaustive exercise endurance, quantified as duration to exhaustion from chase, and (2) aerobic performance, quantified by measuring oxygen consumption rate immediately after chase (i.e. MMR) until recovery back to resting levels (i.e. SMR), allowing estimation of aerobic scope as well as EPOC. We assessed the thermal sensitivity of exhaustive exercise endurance and aerobic performance in cunner following the same acute and acclimation protocols described for experiment 1 but using different fish and with an acclimation time of 5–7 weeks (*n*=11–16; see Table S1 for body masses and lengths). In brief, individual fish were transferred via net immersed in seawater from their respective thermal acclimation tank into the pre-test holding system, where they were either (1) cooled or warmed (3°C h^−1^, using 14°C-acclimated fish) to their test temperature between 26 and 2°C at which they remained for 1 h prior to testing (acutely exposed fish), or (2) kept at their acclimation temperature (14, 11, 8, 5 or 2°C) for 5 h prior to testing (acclimated fish). Then, each fish was transferred via net in seawater to the chase arena: a round bucket (55×42×20 cm, diameter×height×water depth) filled with aerated seawater at the same test temperature. After a 15 min adjustment period in the arena, the fish was manually chased by hand until exhaustion, which was defined as no longer bursting away after five gentle caudal fin pinch attempts, and the time to exhaustion was recorded.

Aerobic performance was determined by estimating SMR and MMR via measurement of oxygen consumption rate (*Ṁ*_O_2__; mg O_2_ h^−1^ kg^−1^) using automated intermittent-closed optical respirometry. Following exhaustion from chase, fish were immediately transferred by hand from the chase bucket into a sealed respirometer (∼160 ml volume, glass container with plastic lid fitted with O-ring) submerged within a larger clear plastic water bath (70×41×16 cm, length×width×depth). Each respirometry trial consisted of four fish run simultaneously in individual respirometers. The water bath was supplied with temperature-controlled (at respective test temperature) seawater from an insulated sump via a submersible pump. Each respirometer was fitted with an oxygen probe to measure the within-chamber temperature-compensated oxygen level using a 4-channel FireSting (PyroScience, Aachen, Germany). The respirometer water was mixed with stir bars separated from the fish by plastic mesh and driven by a 6-channel magnetic stir plate placed underneath the water bath. The flush cycles were controlled by a smaller Eheim 600 pump powered through a commercially available programmable digital timer set to temperature-specific open–close intervals. The flush period was always 3 min (except 2 min at 26°C), which ensured full re-oxygenation of the respirometer water, and the closed period was modified depending on experimental water temperature because the slope of oxygen decline (metabolic rate) was lower at cooler temperatures (5 min for 26 and 23°C, 6 min for 20°C, 7 min for 17°C, 9 min for 14 and 11°C, 17 min for 8°C, 27 min for 5°C, and 42 min for 2°C). Fish were allowed to recover from the chase exercise in the respirometers overnight and were removed the following morning (∼14–18 h total recorded *Ṁ*_O_2__), allowing estimation of MMR, SMR and EPOC. Background respiration rates in the respirometers were calculated after removing each fish and recording oxygen change over three closed cycles. Once background measurements were recorded, the system was cleaned with dilute bleach and rinsed twice with freshwater before re-use.

*Ṁ*_O_2__ was measured from the slope of the decline in water oxygen content during the closed period; the slopes were extracted using LabChart (Version 8.1.13, ADInstruments, Colorado Springs, CO, USA). We calculated metabolic rate using the following equation (Schurmann and Steffensen, 1997):
(1)

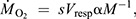
where *s* is the slope (O_2_ % air saturation h^−1^), *V*_resp_ is the volume of the respirometer loop minus the volume of the fish (l), α is the solubility of oxygen in water (mg O_2_ l^−1^ O_2_ % air saturation^−1^) adjusted for temperature and barometric pressure, and *M* is the mass of the fish (kg). The average of the three background respiration cycles per trial per respirometer was used to correct each fish's *Ṁ*_O_2__.

MMR was calculated using the maximum slope of oxygen decline in a 60-s period following exhaustive exercise. This maximum slope was identified by an iterative approach in which 60-s slopes were taken sequentially in 30 s increments from the moment exercised fish were placed in the respirometer (i.e. 0–60, 30–90, 60–120 s, etc.) over the first 15 min of closed periods; the highest slope always occurred within the first 4.5 min post-exercise (with 94% of MMR occurring in the first 2.5 min). Subsequent to the closed period in which MMR occurred, *Ṁ*_O_2__ values were calculated using the entire slope of oxygen decline during each entire closed cycle (excluding the first 120 s and last 60 s to ensure equilibration following flush and avoid sampling the beginning of the flush period) until the fish was removed from the respirometer. SMR was calculated as the average of the lowest 10% slopes of oxygen decline, excluding any outliers (outside 2 standard deviations of the average of the lowest 10% slopes). In the absence of simultaneous measurement of activity, the average of a lowest subset percentage of *Ṁ*_O_2__ values is a common method to estimate SMR in fishes (Chabot et al., 2016). Absolute aerobic scope (AAS) was calculated for each fish by subtracting their SMR from their MMR.

To calculate EPOC and recovery time, *Ṁ*_O_2__ of each fish following its MMR was more finely calculated by taking the slopes of oxygen decline over sequential 60-s periods within each closed period (excluding the first 120 s and last 60 s of a cycle) until 4 h post-MMR; then the oxygen decline was measured over a 3 min period every 40–50 min (depending on the temperature) until the fish was removed from the respirometer the following morning. During the 4-h post-MMR period, we filtered the data to remove *Ṁ*_O_2__ values at time points where *Ṁ*_O_2__ values were not available for all fish (at certain time points, some fish were in a flush period and thus without a measured *Ṁ*_O_2__, because fish were chased and introduced to their respirometers over time within a given closed cycle). Thus, a *Ṁ*_O_2__ recovery curve was generated for each fish. Recovery time was defined as the time post-MMR at which the first of three consecutive *Ṁ*_O_2__ values were equal to SMR±10%. Total EPOC for each fish was determined by integrating the area between the recovery curve and SMR, i.e. from the time of MMR to the recovery time (Lee et al., 2003).

We also estimated the exhaustive exercise effort (BL swum until exhaustion) of the EPOC fish at each temperature using the following equation:
(2)

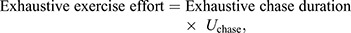
where exhaustive chase duration (s) is the mean at each temperature for the fish manually chased to subsequently measure total EPOC (experiment 2), and *U*_chase_ (BL s^−1^) is the mean at the same temperature for the fish in the annular swim test (experiment 1) as the best available proxy for the swim speed of the EPOC fish during their own chase (i.e. *U*_chase_ had not been measured in experiment 2). Mean values were used because chase duration and *U*_chase_ were necessarily from different experiments and thus from different individual fish, so it was not possible to calculate individual effort values. We then modelled the relationship between exhaustive exercise effort and total EPOC to examine whether EPOC is simply an outcome of the exhaustive exercise effort possible at a given temperature (which would be indicated by a strong relationship), or whether constraints of cold on EPOC processes or their coupling to exercise effort occur (which would be suggested by a poor relationship). We calculated and used exhaustive exercise effort for this purpose because: (1) there could be different temperature-dependent effects on intensity (*U*_chase_) or duration of exercise (e.g. fish could be more likely to swim faster but for a shorter time at warmer versus colder temperatures, with potentially distinct disturbances or costs from exercise in each case), and (2) EPOC was measured in experiment 2 alongside chase duration only (not *U*_chase_), but chase duration does not account for exercise intensity's potential influence on EPOC; because *U*_chase_ was measured on different fish than EPOC, the product of chase duration and *U*_chase_ is the best approximation of the exercise effort of the fish in which EPOC was measured. Overall, exhaustive exercise effort is the best integrated measure of exercise's energetic burden for the fish to move its body until exhaustion from chase, and which must be recovered from during EPOC.

### Experiment 2B: muscle metabolic responses

#### Tissue collection

Additional cunner acclimated for 7–8 weeks to 14, 8 and 2°C during experiment 2A were exhaustively chased following the same protocol described in experiment 2A, but instead of being transferred to a respirometer, were killed via cervical dislocation (*n*=10 per temperature; see Table S1 for body masses and lengths) for measurement of energy metabolite contents in white muscle. Following euthanasia and body size measurement, a white muscle steak (∼1 cm thick, anterior of the caudal peduncle) was flash-frozen in liquid nitrogen. Additionally, non-exercised fish that had been either acutely cooled or acclimated for 7–8 weeks to 14, 8 and 2°C were sampled in the same manner to serve as resting controls for metabolite measurements. Measurements of metabolic enzyme activities were also made on chased fish from each acclimation temperature. Muscle was stored in a −80°C freezer, then ground under liquid nitrogen with a mortar and pestle and stored at −80°C until enzyme and metabolite assays. All chemicals used in the enzyme and metabolite assays were purchased from Sigma-Aldrich (St Louis, MO, USA).

#### Enzyme activities

Frozen ground white muscle (47–60 mg) was homogenized in ice-cold homogenization buffer (50 mmol l^−1^ imidazole, 1 mmol l^−1^ disodium EDTA, 0.1% Triton X, pH 7.4) using a polytron-type homogenizer (BioSpec Products, Bartlesville, OK, USA) with three 5-s bursts at the highest speed setting. We added 15 volumes of buffer when samples weighed <25 mg and 10 volumes of buffer when samples weighed >25 mg. Homogenates were spun in a microcentrifuge at 1000 ***g*** for 5 min at 4°C. The supernatant was diluted and used to measure the activities of lactate dehydrogenase (LDH), citrate synthase (CS) and creatine kinase (CK). We measured LDH activity to assess anaerobic glycolytic capacity, CS activity as an indicator of aerobic capacity, and CK activity given its importance for rapid substrate-level generation of ATP during burst exercise (Guderley et al., 2001).

We measured maximal enzyme activities at a common temperature (25°C) with a SpectraMax 190 microplate spectrophotometer (Molecular Devices, Sunnyvale, CA, USA) by following the oxidation or reduction of pyridine dinucleotides at 340 nm [millimolar coefficient 6.22 (mmol l^−1^)^−1^ cm^−1^] (LDH and CK) or the appearance of 5-thio-2-nitrobenzoic acid resulting from the reaction of liberated free CoA with 5,5′-dithiobis (2-nitrobenzoic acid) (DTNB) at 412 nm [millimolar coefficient 13.6 (mmol l^−1^)^−1^ cm^−1^] (CS) over a 10-min reaction period. The assay conditions were as follows (in mmol l^−1^): LDH=50 imidazole, pH 7.4, 0.2 NADH and 1 pyruvate (omitted for control); CK=50 imidazole, pH 7.4, 1 ADP, 10 AMP, 0.3 NADP, 4 d-glucose, 5 MgCl_2_, 2 U ml^−1^ hexokinase, 2 U ml^−1^ glucose-6-phosphate dehydrogenase and 25 creatine phosphate (omitted for control); and CS=50 imidazole, pH 7.4, 0.3 acetyl-CoA, 0.1 DTNB and 0.5 oxaloacetate (omitted for control). Substrates were at saturating levels and optical pathlength was calculated for a final microplate well volume of 200 µl. Enzyme activity was measured in duplicate and run simultaneously with a single control reaction with omitted substrate to correct for non-specific background reactions. Preliminary measurements confirmed the reaction rates to be linear with time and proportional to the amount of homogenate added.

Enzyme activities were calculated as nmol substrate converted to product per minute per milligram tissue protein (nmol min^−1^ mg^−1^ protein) as well as µmol substrate converted to product per minute per gram wet weight (µmol min^−1^ g^−1^ ww). Enzyme activities per gram wet weight indicate the physiologically relevant enzymatic capacity of the tissue, while enzyme activities per milligram protein allow us to correct for potential influences of variable tissue protein content. Protein content of the enzyme supernatant was measured using the Bradford assay.

#### Metabolite contents

Approximately 90 mg of frozen ground muscle was homogenized in 10 volumes of 8% perchloric acid using three 5-s bursts of a polytron-type homogenizer (BioSpec Products) at its highest setting. Neutralized extracts were thawed on ice and assayed for ATP, creatine phosphate, lactate and glycogen according to well-established protocols (Bergmeyer, 1983).

### Thermal sensitivity analysis

The thermal sensitivity of each performance rate metric measured in experiments 1 and 2A was calculated as the temperature coefficient, *Q*_10_:
(3)

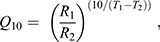
where *R* is the performance rate at the respective temperature *T*. Specifically, we calculated *Q*_10_ for both acutely exposed and acclimated fish across the temperature intervals that bracket the WDTT (14–8°C and 8–2°C), and if our hypothesis was supported, we expected to observe a greater constraint (higher *Q*_10_, specifically >3) below the WDTT than above, regardless of acclimation history. We also calculated *Q*_10_ for the 14–20°C acute exposure interval; cunner commonly experience up to 20°C during summer activity and so we expected this *Q*_10_ to be similar to that for the 14–8°C interval, which also represents temperatures of normal activity (i.e. above the WDTT) (Reeve et al., 2022). To further assess how cooling constrains cunner performance, we also calculated the percent change of mean performance over the same temperature intervals for every metric. The analysis of percent change in performance was useful, in particular, to quantify the thermal sensitivity of the non-rate performance metrics (i.e. not expressed in per unit time: C-start responsiveness, exhaustive chase duration and EPOC recovery time) where *Q*_10_ analysis is inappropriate. Because individual fish were only ever tested at one temperature to avoid effects of repeated testing and sequential temperature exposures, we used the mean performance value of all fish at each relevant temperature to calculate a single *Q*_10_ or percent change value for each performance metric and interval, precluding statistical analysis on the thermal sensitivities within each metric. However, we calculated a mean ‘omnibus’ performance *Q*_10_ or percent change value for each aforementioned temperature interval within the acute or acclimated treatments by averaging the corresponding *Q*_10_ values of all performance rate metrics or percent change values of all performance metrics. This allowed us to statistically investigate (see below) whether the omnibus thermal sensitivity (i.e. mean *Q*_10_ or percent change) of multiple metabolic and exercise performance metrics varies with acute and acclimated temperature change above and below the WDTT. For example, a significant increase in the omnibus *Q*_10_ following acute and acclimated cooling below the WDTT would support our hypothesis. Note, although values for *Q*_10_ and percent change of SMR were calculated, these were excluded from the above analyses because SMR is a basic cost of maintenance, not performance per se.

### Statistics

We used R for all analyses (version 4.0.2, www.r-project.org). Assumption of normality was tested by examining normality plots and homogeneity of variances were tested using Levene's test of equality of variances. Statistical significance was accepted at *P*<0.05 and all results values are presented as means±s.e.m., unless otherwise stated.

The effects of temperature (14, 11, 8, 5 or 2°C), acclimation status (acutely exposed or acclimated) and their interaction on each performance metric were tested using a two-way ANOVA with Tukey HSD *post hoc* tests. These two-way ANOVAs of acute and acclimated performance between 14 and 2°C evaluated the constraints of cold on performance and tested our prediction that dormant cunner would lack compensation via plasticity (i.e. no difference between the acute and acclimated performance level at a given temperature). Additional acute temperature exposures above 14°C were also carried out; the effect of acute exposure to temperature (26, 23, 20, 17, 14, 11, 8, 5 or 2°C) on each performance metric was separately assessed using a one-way ANOVA. These one-way ANOVAs of acute thermal performance were used to characterize the performance of cunner over a broader temperature range, including approximation of optimal temperatures. The effect of acute and acclimated temperature interval (acute 14–20°C, acute 14–8°C, acute 8–2°C, acclimated 14–8°C and acclimated 8–2°C) on the mean *Q*_10_ of all performance rate metrics and the mean percent change of all performance metrics was determined using individual one-way ANOVAs and Tukey HSD *post hoc* tests. The relationship between exhaustive exercise effort and total EPOC was determined using linear regressions for both acclimated and acute groups from 14 to 2°C, as that was the temperature range shared between acclimated and acute exposures. The effect of acclimation temperature (14, 8 and 2°C) on LDH, CS and CK activity in white muscle of cunner was tested using one-way ANOVAs and Tukey HSD *post hoc* tests. The effects of temperature (14, 8 and 2°C), exercise treatment (acute resting, acute post-exercise, acclimated resting and acclimated post-exercise), and their interaction on white muscle metabolite contents were tested using individual two-way ANOVAs and Tukey HSD *post hoc* tests for each parameter.

## RESULTS

**Fig. 2.**
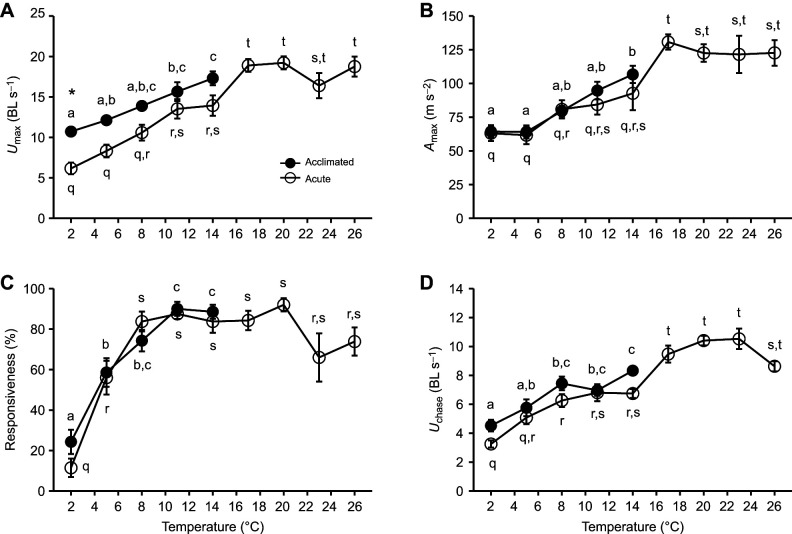
**Effects of acute temperature change (3°C** **h^−1^ using 14°C-acclimated animals) or temperature acclimation (5–6 weeks) on escape swimming responses in cunner.** Data are shown for C-start test variables (A) maximum burst velocity (*U*_max_), (B) maximum burst acceleration (*A*_max_) and (C) responsiveness (% of successful C-starts from five C-start stimuli) (see Materials and methods), as well as (D) annular chase swimming speed (*U*_chase_) measured in a subsequent test on the same fish (laps swam in a circular raceway over time during chase until exhaustion) (experiment 1). Data are means±s.e.m. (*n*=see Table 1). Asterisks indicate a significant difference between acclimation and acute values at the same temperature. Dissimilar letters indicate values with significant differences between temperatures within the acute or acclimated exposures (two-way ANOVA for acclimated and acute responses between 14 and 2°C; one-way ANOVA for acute responses from 26 to 2°C with Tukey HSD *post hoc* tests, *P*<0.05).

**Fig. 3.**
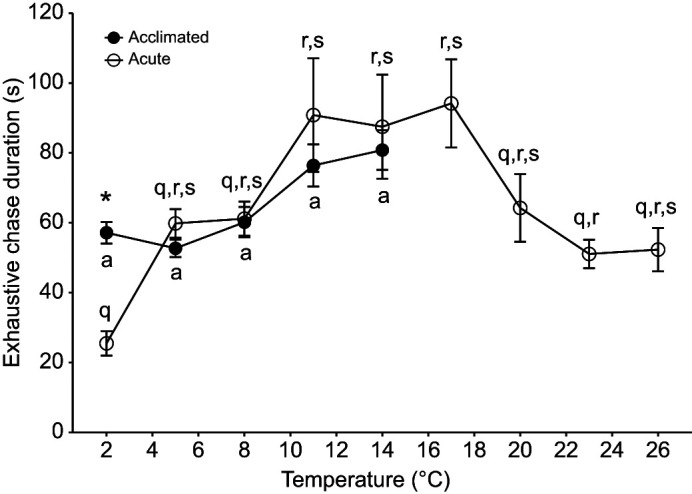
**Effects of acute temperature change (3°C** **h^−1^ using 14°C-acclimated animals) or temperature acclimation (5–7 weeks) on exhaustive exercise endurance (time to exhaustion from manual chase) in cunner (experiment 2A).** Data are means±s.e.m. (*n*=see Table 1). See Fig. 2 for statistical details.

**Fig. 4.**
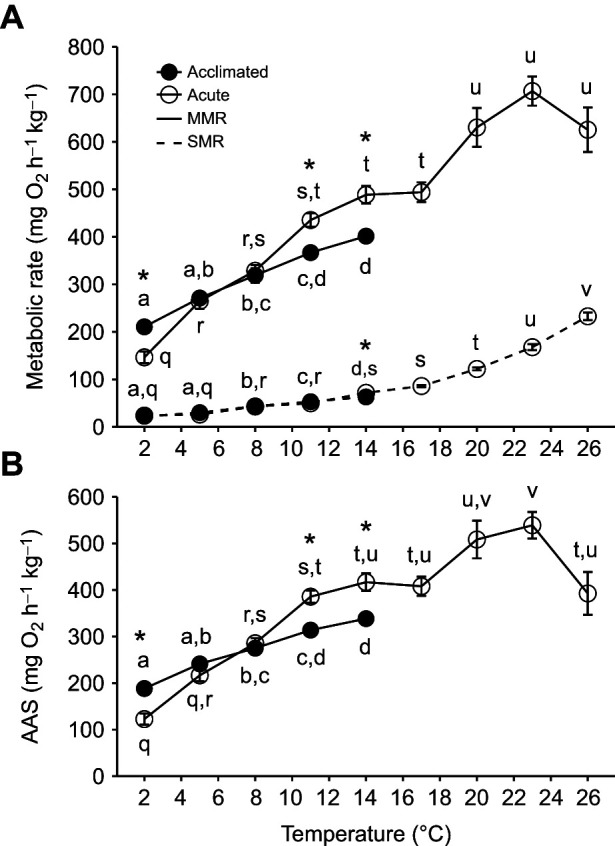
**Effects of acute temperature change (3°C** **h^−1^ using 14°C-acclimated animals) or temperature acclimation (5–7 weeks) on aerobic performance in cunner (experiment 2A).** (A) Standard and maximum metabolic rates (SMR and MMR), and (B) absolute aerobic scope (AAS). Data are means±s.e.m. (*n*=see Table 1). See Fig. 2 for statistical details.

**Fig. 5.**
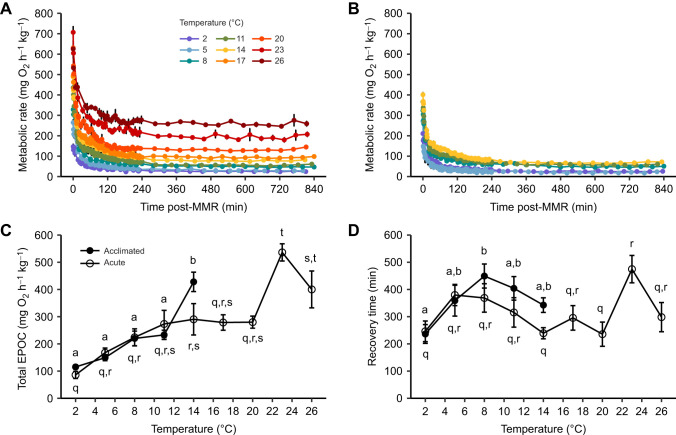
**Effects of acute temperature change (3°C** **h^−1^ using 14°C-acclimated animals) or temperature acclimation (5–7 weeks) on excess post-exercise oxygen consumption (EPOC) in cunner (experiment 2A).** Metabolic rate following exhaustive exercise is shown over a 14-h recovery period from MMR to resting for (A) acutely exposed animals and (B) acclimated animals. Also shown are (C) total EPOC and (D) the time to recovery of metabolic rate to ±10% of SMR (see Fig. 4A). Data are means±s.e.m. (*n*=see Table 1). See Fig. 2 for statistical details; note, here acclimation and acute values were not significantly different at any same exposure temperature.

**Fig. 6.**
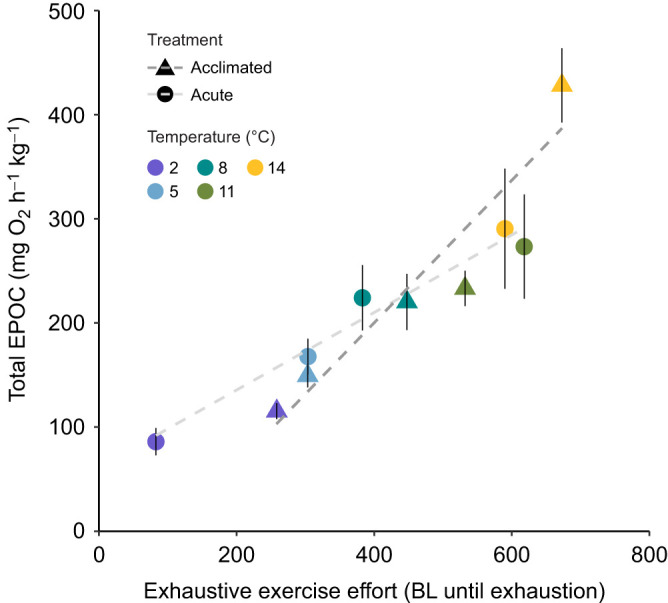
**Relationship between mean exhaustive exercise effort and mean total EPOC of cunner following acute temperature change (3°C** **h^−1^ using 14°C-acclimated animals) (closed circles) or acclimation (5–7 weeks) (closed triangles) to 14, 11, 8, 5 or 2°C.** Exhaustive exercise effort was calculated as exhaustive chase time from experiment 2A fish×annular chase swim speed from experiment 1 fish, hence mean values for each parameter had to be used and individual values for effort could not be calculated precluding inclusion of error bars for effort. Total EPOC (±s.e.m.) are for experiment 2A fish. Linear regression lines are fitted across the common temperature range of 14–2°C for acclimated (dark grey; *y*=0.6833*x*–78.25, *R*^2^=0.91, *P*=0.012) or acute (light grey; *y*=0.376*x*+56.02, *R*^2^=0.97, *P*=0.002) exposures; the slopes differ significantly (*P*<0.05).

**Fig. 7.**
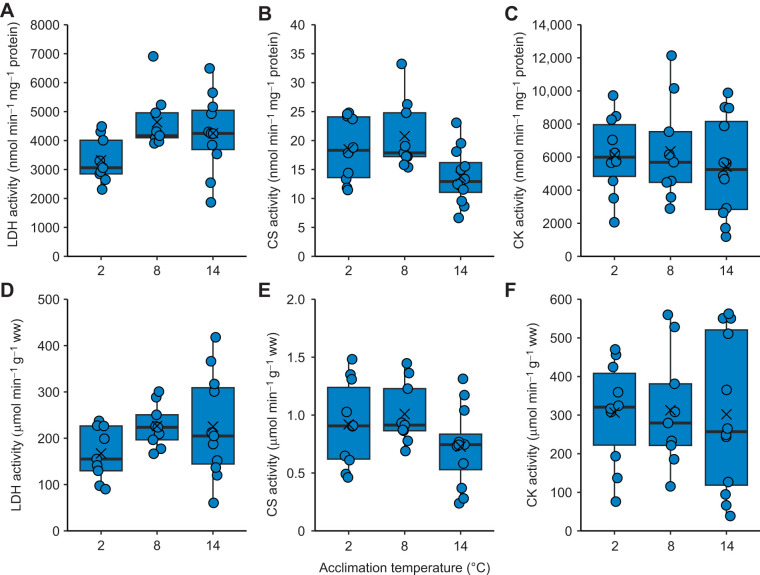
**Effect of temperature acclimation (7–8 weeks) on white muscle metabolic enzyme activities in cunner (experiment 2B).** Lactate dehydrogenase (LDH), citrate synthase (CS) and creatine kinase (CK) activities are shown as nmol min^−1^ mg^−1^ protein (A–C) or µmol min^−1^ g^−1^ ww (D–F). Box plot elements: ×, mean; horizontal line, median; box limits, 25th and 75th percentiles; whiskers, percentiles±IQR×1.5; circles, individual data values (for *n*-values, see Table 1). Enzyme activities were unaffected by temperature (one-way ANOVA, *P*>0.05).

**Fig. 8.**
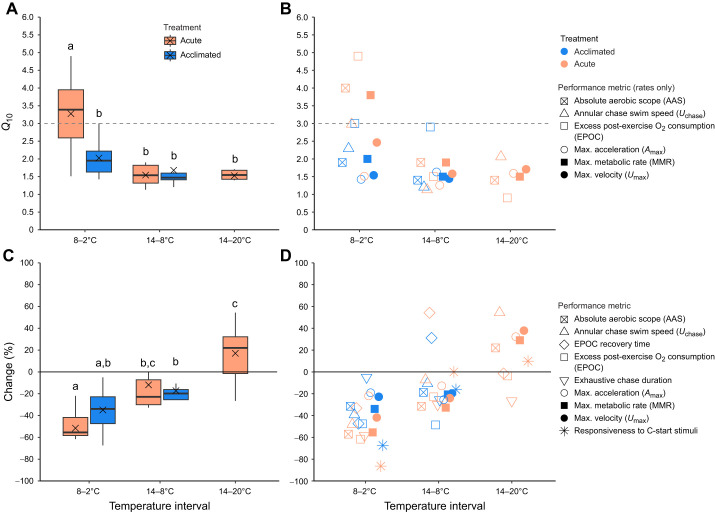
**Thermal sensitivity of cunner performance following acute temperature change (3°C** **h^−1^ using 14°C-acclimated animals) (orange symbols) or acclimation (5–7 weeks) to 14, 8 or 2°C (blue symbols).** (A) Box plot summaries of the (B) *Q*_10_ values of performance metrics associated with the change in the mean value of each performance metric (excluding non-rate performance, i.e. exhaustive chase duration, EPOC recovery time, responsiveness) across 6°C intervals above and below the winter dormancy threshold temperature (7–8°C for cunner; Reeve et al., 2022), as well as following acute warming of 14°C-acclimated animals to 20°C (i.e. 14–20°C, 14–8°C: normal activity; 8–2°C: dormant). *Q*_10_ values above 3 (dashed grey line) infer a thermal constraint (see Fig. 1). (C) Box plot summaries of the (D) percent changes in the mean value of each performance metric across the same temperature intervals as for *Q*_10_. To reiterate, the box plots in A and C were generated using the individual performance *Q*_10_ or percent change data in C and D for the corresponding temperature interval and acute or acclimated treatment. The solid black line at *y*=0 indicates thermal independence, i.e. unchanged performance across the temperature interval. Acclimated data are lacking for 14–20°C because fish were not acclimated to >14°C. Different groups of cunner were used for each acute or acclimated temperature exposure, so we used mean performance for each calculation. Dissimilar letters indicate significant differences between the mean *Q*_10_ or percent change values in A or C (one-way ANOVAs with Tukey *post hoc* tests, *P*<0.05). Box plot elements: ×, mean; horizontal line, median; box limits, 25th and 75th percentiles; whiskers, percentiles±IQR×1.5. See Table S4 for the plotted numerical values.

The responses of the measured variables to acute or acclimated temperature exposures are shown in Figs 2–7; the corresponding thermal sensitivities of performance above and below the WDTT are shown in Fig. 8.

**Table 1.**
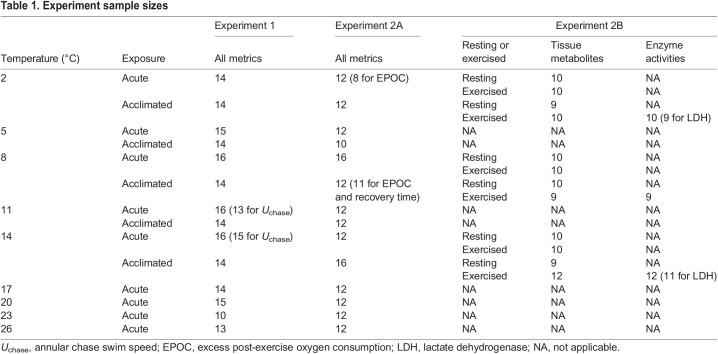
Experiment sample sizes

### Experiment 1: swim performance

*U*_max_ and *U*_chase_ significantly increased with acute warming of 14°C-acclimated cunner, and a plateau of maximum performance was reached from 17 to 23°C (i.e. a broad *T*_opt_) (*P*<0.0001; Fig. 2). Cooling from 14 to 2°C caused significant reductions in swim speeds of both acutely exposed and acclimated cunner (*P*<0.0001; Fig. 2); chase duration and distance swam during the annular swim showed a similar response (Table S3). *U*_chase_ was similar between acute and acclimated fish at every temperature (Fig. 2D). *U*_max_ was greater on average in acclimated cunner compared with acutely cooled cunner at the same temperature, but this difference was only significant at 2°C, where acclimated *U*_max_ was nearly twice as high as acute *U*_max_ (Fig. 2A). Acutely exposed and acclimated cunner had similar *A*_max_ and responsiveness at all temperatures from 14 to 2°C (Fig. 2B,C). *A*_max_ decreased modestly with cooling and was similarly temperature dependent above and below the WDTT for both acutely cooled and acclimated fish (*P*<0.0001; Figs 2B and 8B; also see Fig. S1). *U*_max_ and *U*_chase_ following acute temperature change were more thermally sensitive below the WDTT (8–2°C; *Q*_10,acute8–2°C_=2.5 and 3.0, respectively) than above (*Q*_10,acute14–8°C_=1.6 and 1.1, respectively), but partial compensation accompanied 2°C acclimation (*Q*_10,acclimated8–2°C_=1.5 and 2.3 for *U*_max_ and *U*_chase_, respectively) (Fig. 8B, Table S4; also see Fig. S1). In contrast, responsiveness plummeted from ∼80% to ∼20% successful C-starts between 8°C and 2°C, with little improvement following acclimation (i.e. −86.4% versus +0.0% acute change or −67.3% versus −16.1% acclimated change for 8–2°C versus 14–8°C, respectively) (*P*<0.0001; Figs 2C and 8D, Table S4).

### Experiment 2A: exhaustive exercise endurance and aerobic performance

Exhaustive exercise endurance (i.e. exhaustive chase duration; Fig. 3) and aerobic performance metrics (Fig. 4) significantly decreased with cooling below their *T*_opt_ range in both acclimated and acutely exposed cunner (*P*<0.0001). Based on the plateaus of maximum performance of acutely exposed cunner, the *T*_opt_ range was approximately ∼11–17°C for exhaustive exercise endurance and 20–23°C for aerobic performance. At acute exposure temperatures warmer than *T*_opt_, chase duration and aerobic performance decreased. SMR consistently decreased with temperature (*Q*_10_ of 1.8–3.1) and was similar between acclimated and acute fish at each temperature (Fig. 4A, Table S4). The percent change in exhaustive chase duration and the thermal sensitivities of MMR and AAS in cunner acutely exposed to temperature change were greater below the WDTT than above the WDTT (*Q*_10,acute8–2°C_=3.8–4.0 versus *Q*_10,acute14–8°C_=1.9 and *Q*_10,acute14–20°C_ =1.4–1.5) (Figs 8B,D, Table S4; also see Fig. S1). Above the WDTT, acclimated and acutely cooled fish had similar temperature dependency for exhaustive chase duration, MMR and AAS (*Q*_10,acclimated14–8°C_=1.4–1.5) (Fig. 8B,D, Table S4). At 2°C, but at no warmer acclimation temperature, exhaustive chase duration, MMR and AAS of acclimated fish was significantly higher compared with performance of acutely cooled fish (*P*<0.001; Figs 3 and 4). This occurred despite the MMR and AAS of acclimated cunner at 11 and 14°C happening to be significantly lower than acute cunner (*P*<0.01); furthermore, at 5 and 8°C, MMR and AAS were not different between acclimated and acute fish, and chase duration was not different between acute and acclimated fish at any temperature except 2°C (*P*>0.05; Figs 3 and 4). Below the WDTT (8–2°C), acclimated cunner had a much smaller constraint on exhaustive chase duration (Fig. 8D, Table S4) and markedly lower *Q*_10_ values (similar to those above the WDTT) for MMR and AAS compared with acutely cooled cunner (*Q*_10,acclimated8–2°C_=1.9–2.0 versus *Q*_10,acute8–2°C_=3.8–4.0) (Fig. 8B, Table S4).

Following exhaustive chase, the metabolic rates of fish acclimated or acutely exposed to each temperature were elevated before gradually recovering to SMR±10% by 3.9–7.9 h post-MMR (Fig. 5A–D). Total EPOC decreased with cooling below 11–14°C (while being unusually elevated at the two warmest acute temperature exposures), but recovery time was relatively similar across temperatures; both were statistically similar between acclimated and acutely exposed cunner at all temperatures (Fig. 5C,D). Exhaustive exercise effort (exhaustive chase time×*U*_chase_) was significantly positively correlated with total EPOC in both acutely cooled and acclimated cunner across their common test temperatures of 14–2°C; the slopes differed significantly from one another (Fig. 6). The thermal sensitivity of total EPOC above and below the WDTT showed a similar pattern to that described above for MMR and AAS (Fig. 8B, Table S4; also see Fig. S1).

### Experiment 2B: muscle metabolic responses

LDH and CK enzyme activities were not significantly affected by acclimation temperature, regardless of whether expressed as a function of protein content or tissue capacity (Fig. 7A,D and C,F). CS enzyme activity also was unaffected by temperature, although in 2°C-acclimated fish it was 75–79% higher on average compared with 14°C-acclimated fish (Fig. 7B,E).

Exhaustive chase durations of fish used for metabolite measurements in experiment 2B (Fig. S2A) were similar to those for experiment 2A (Fig. 3), including similar effects of acute and acclimated temperature exposures (specifically, significant compensation at 2°C). Temperature and the treatment×temperature interaction significantly affected glycogen content (*P*<0.0001) (Fig. S2B). Resting glycogen was significantly lower in cunner acclimated to 2°C compared with 8 or 14°C (*P*=0.01 or 0.03, respectively). Glycogen levels were similar pre- and post-exercise at all acute or acclimated temperatures, but showed a decreasing trend following exercise at 14°C, likely explaining the temperature×treatment interaction. Lactate content was significantly affected by temperature, treatment and their interaction (*P*<0.0001, 0.0001 and 0.001, respectively) (Fig. S2C). Resting lactate levels were similar across temperature and treatment groups. In acutely cooled cunner, mean lactate contents were higher post-exercise compared with rest at every temperature, but only significantly at 14°C (*P*=0.002). In acclimated cunner, exhaustive exercise increased lactate content at 14 and 8°C, but not 2°C (*P*<0.0001 and *P*=0.048 for 14 and 8°C, respectively). Exhaustive exercise significantly affected creatine phosphate (CrP) stores (*P*<0.0001) (Fig. S2D). CrP levels were always lower following exercise, although only significantly in 2°C-acclimated cunner (*P*=0.013). Resting and post-exercise CrP contents were unaffected by temperature or acute/acclimated exposure.

## DISCUSSION

Our comprehensive analysis of thermal exercise and metabolic performance of cunner did not support the cold performance limitation hypothesis for winter dormancy. Instead, we revealed for the first time that partial thermal compensation of performance via acclimation occurs even in a winter-dormant fish. As is typical for ectotherms (Johnston and Temple, 2002; Schulte, 2015), the burst escape, exhaustive exercise and aerobic performance of cunner declined with cooling below their respective thermal optima (∼17–23°C for cunner based on our acute exposures). Acute exposure or acclimation to temperatures above the WDTT of 7–8°C (Reeve et al., 2022) was associated with comparable, typical *Q*_10_ values for teleost performance rates of 1–3 (*Q*_10,acute14–8°C_=1.1–1.9, mean=1.5; *Q*_10,acute14–20°C_=0.9–2.1, mean=1.5; *Q*_10,acclimated14–8°C_ =1.2–2.9, mean=1.7) (Fig. 8A,B) (Clarke, 2017; Jørgensen et al., 2022; Seebacher et al., 2015). Below the WDTT, the acute thermal sensitivities of performance were significantly higher (*Q*_10,acute8–2°C_ range=1.5–4.9, mean=3.3), indicating a thermal constraint from rapid cooling consistent with the cold performance limitation hypothesis. However, contrary to our hypothesis, performance never neared zero and actually was enhanced following multi-week acclimation at 2°C, abolishing the acute constraint and resulting in lowered *Q*_10_ values not significantly different from the typical *Q*_10_ values across active temperatures above the WDTT (*Q*_10,acclimated8–2°C_=1.4–3.0, mean=2.0) (Fig. 8A). Analysis of the percent decreases of all performance metrics supported the *Q*_10_ analysis, with a greater acute constraint below the WDTT (8–2°C) that was blunted following 2°C-acclimation (Fig. 8C,D). Thus, our findings indicate partial thermal compensation and relatively unconstrained performance in cunner when acclimated to a typical, dormant winter temperature of 2°C. Because acclimation has a prolonged timescale more relevant to the gradual seasonal cooling associated with winter onset for wild cunner (Costa et al., 2013), our results provide strong evidence against the cold performance limitation hypothesis. Overall, winter dormancy in a model species, the cunner, does not appear to operate as a behavioural filter (Garland and Losos, 1994) to mitigate thermal constraints on metabolic or exercise physiology.

### Thermal responses of burst escape and exhaustive exercise performance

The C-start burst escape response (e.g. *U*_max_) and exhaustive exercise performance (e.g. *U*_chase_ and exhaustive chase duration) are critical for predator evasion and foraging success in fishes (Marras et al., 2011; Videler and Wardle, 1991). In cunner, *U*_chase_ and exhaustive chase duration were constrained (*Q*_10_>3) by acute cooling below the WDTT, but were strongly compensated following 2°C acclimation and otherwise had *Q*_10_ typical for swimming (e.g. *U*_crit_) and metabolic-linked performance traits in fishes (i.e. 1–3). *U*_chase_ and exhaustive chase duration have been rarely measured in fishes, limiting interspecific comparison of their thermal sensitivities. *U*_max_ and *A*_max_ in cunner had low thermal sensitivity (*Q*_10_=1.3–1.6) in acclimated animals or following acute temperature change above the WDTT, which is typical for acclimated or acutely exposed fishes, including in tropical, winter-active and winter-lethargic species, as well as a previous study on cunner (Beddow et al., 1995; Claireaux et al., 2006; Johnson et al., 1996; Moran et al., 2019; O'Steen and Bennett, 2003; Temple and Johnston, 1998). Acute cooling of cunner below the WDTT was associated with a greater thermal constraint on *U*_max_ (*Q*_10,acute8–2°C_=2.5); similarly, *Q*_10_ values >2 have been observed in tropical damselfishes acclimated to stressful temperatures cooler than their latitudinal range limit and in cold temperate sculpins acutely exposed to an unusually frigid 0.8°C temperature (Djurichkovic et al., 2019; Temple and Johnston, 1998). Thus, extreme cold exposure can constrain C-start performance in fishes regardless of overwintering strategy. Importantly, compensation of cunner *U*_max_ enabled robust cold performance following winter-relevant 2°C acclimation. Compensatory thermal plasticity of the C-start escape response is common among cold-active fishes (Johnston and Temple, 2002), but here we show that this extends to a winter-dormant fish, alongside compensation of exhaustive exercise performance.

The C-start also involves important non-locomotor components (e.g. responsiveness to a predator) (Marras et al., 2011). Unlike the C-start locomotor components (e.g. *U*_max_), responsiveness to the C-start stimulus in cunner was greatly constrained by cold and was uncompensated following acclimation. Extreme cold can cause impaired sensorimotor function and lethargy in fishes, but usually only as they reach their lower critical limit or following large acute cooling (Friedlander et al., 1976; Preuss and Faber, 2003; Reid et al., 2022). C-start latency (time between stimulus and C-start) has been found to increase with cooling in fishes (Morgan et al., 2022; Preuss and Faber, 2003), but little is known about thermal effects on responsiveness to stimuli. This is important because not responding to a stimulus can have greater repercussions than a small millisecond change in the latency of a successful C-start. Two previous studies provide comparisons to our results for cunner. First, Johnson and Bennett (1995) reported that only 30% of 35°C-acclimated goldfish responded to C-start stimulus after acute 10°C exposure. Second, following acute exposure of 15°C-acclimated sculpins to 0.8°C, Temple and Johnston (1998) reported that C-start responsiveness of the Arctic-ranging *Myoxocephalus scorpius* was unaffected, whereas only 40% of the temperate *Taurulus bubalis* responded; following 5°C acclimation, however, all *T. bubalis* responded. Thus, cunner are conspicuous in the constraint that winter cold has on their C-start responsiveness, which could elevate predation risk if not for their inactive, sheltering dormancy. Interestingly, all cunner C-started in response to direct touch, even at 2°C, hinting that cold specifically constrains visual or mechanoacoustic senses. Unlike the other escape response metrics, the great cold sensitivity and limited compensation of responsiveness, and underlying sensory pathways, could be an alternative proximate explanation for winter dormancy in fishes.

### Thermal responses of aerobic performance

MMR and AAS of cunner were constrained by acute cooling below the WDTT (*Q*_10_=3.8–4.0 from 8 to 2°C versus 1.4–1.9 above 8°C) but were strongly compensated following cold acclimation (*Q*_10,acclimated8–2°C_=1.9–2.0), contradicting our hypothesis. In fact, even at dormant temperatures, the acclimated thermal sensitivity of aerobic performance in cunner was similar to that of winter-active fishes, in which acclimated *Q*_10_ values fall within the typical range of 1–3 across species-specific summer and winter temperatures (Brett, 1964; Hvas et al., 2018; Mackey et al., 2021; Porter and Gamperl, 2023; Schurmann and Steffensen, 1997; Wolfe et al., 2020). Furthermore, the absolute levels of acclimated MMR and AAS were robust in cunner even at 2°C, being similar to mass- and temperature-corrected rates measured at 2–5°C in non-dormant species with comparable athleticism (Atlantic cod, *Gadus morhua*; Atlantic killifish, *Fundulus heteroclitus*) (Claireaux et al., 2000; Healy and Schulte, 2012; Reeve et al., 2022; Schurmann and Steffensen, 1997; Tirsgaard et al., 2015). Little is known about thermal dependency of aerobic performance in winter-dormant species; however, the winter-lethargic Atlantic killifish, like cunner, exhibited constrained aerobic performance with acute cooling and partial compensation following cold acclimation (thus, lowering thermal sensitivity; AAS *Q*_10,acute15–10°C_=3.5 and *Q*_10,acute10–5°C_=4.4 versus *Q*_10,acclimated15–10°C_=2.1 and *Q*_10,acclimated10–5°C_=2.3) (Healy and Schulte, 2012). Although MMR and AAS at 11°C and 14°C in cunner were greater in acutely exposed fish compared with acclimated fish, likely a result of interindividual variability similarly observed in another study with a comparable design (Healy and Schulte, 2012), the acclimated fish still had greater MMR and AAS at 2°C, highlighting the compensatory cold acclimation. Overall, contrary to our hypothesis, cold sensitivity of aerobic performance is similar between winter-active and winter-dormant or -lethargic fish, because of a shared ability for partial compensation via plasticity.

SMR *Q*_10_ in cunner was ∼2–3, confirming that energy savings in winter-dormant fishes primarily arise from inactivity and passive thermodynamic slowing of SMR, and not from metabolic rate depression (Crawshaw, 1984; Reeve et al., 2022; Speers-Roesch et al., 2018).

### Thermal responses of metabolic enzyme activities

The partial cold compensation of whole-animal exercise and metabolic performance was not associated with compensatory increases of white muscle metabolic enzyme activities. However, neither was there a decrease in enzyme activities, consistent with the non-plastic SMR during dormancy. White muscle CK activity was similar between warm- and cold-acclimated cunner, as found in the putatively winter-lethargic three-spined stickleback (Guderley et al., 2001; Ressel et al., 2022), whereas winter-active species (e.g. chain pickerel) have shown compensation (Kleckner and Sidell, 1985). LDH activity tends to decrease or remain unaffected in white muscle of cold-acclimated fishes (Guderley and Gawlicka, 1992; Guderley et al., 2001; Kleckner and Sidell, 1985), similar to our findings in cunner (perhaps the high activity of white muscle LDH makes compensation unnecessary). Other metabolic adjustments must underlie the partial cold compensation of cunner exhaustive exercise endurance. The unapparent cold compensation of white muscle CS activity in cunner contrasts with findings for winter-active (e.g. rainbow trout) and -lethargic (e.g. smallmouth bass) fishes (Battersby and Moyes, 1998; Guderley and Gawlicka, 1992; Guderley et al., 2001; Kleckner and Sidell, 1985; Kolok, 1991; Orczewska et al., 2010). However, whereas dormant cunner are inactive and non-feeding, winter-lethargic species can maintain low levels of activity and feeding (Reeve et al., 2022; Shuter et al., 2012), potentially explaining their enzymatic compensation. Nonetheless, the response of muscle CS activity to cold acclimation can vary even within a winter-lethargic species, the Atlantic killifish, including increases or no effect (Dhillon and Schulte, 2011; Healy et al., 2017). Thus, cold-induced CS responses may be under a dynamic range of influences, as suggested for cytochrome *c* oxidase (Bremer and Moyes, 2011). Perhaps, the compensation of aerobic performance in cold-acclimated cunner relies more on oxygen supply (e.g. cardiac) adjustments (Keen et al., 2017).

### Thermal responses of exercise recovery

Total EPOC was constrained by acute cooling below the WDTT (*Q*_10_=4.9) whereas temperature change above the WDTT or cold acclimation were associated with *Q*_10_ values (0.9–3.0) typical for fishes (e.g. 1.7–2.4 in salmonids), indicating a partial thermal compensation of EPOC consistent with the partial compensation of the exhaustive chase duration that preceded EPOC measurement (Brett, 1964; Lee et al., 2003). The strong relationship between total EPOC and exhaustive exercise effort (=*U*_chase_×exhaustive chase duration) in acute and acclimated animals suggests that the thermal sensitivity of EPOC in cunner is determined largely by the level of exhaustive exercise achievable at given temperatures; in other words, lowered EPOC with cooling follows from the temperature sensitivity of exhaustive exercise performance rather than that of EPOC-specific processes. This is further supported by the slopes of effort versus EPOC being significantly different between acute and acclimated cunner, probably, in part, because of the enhanced MMR and AAS seen specifically following 2°C acclimation, which would allow increased exercise effort while avoiding a concomitant increase in EPOC (seen as a rightward shift of the 2°C value following acclimation in Fig. 6). This scenario is also consistent with the lack of lactate accumulation at 2°C compared with increases at other temperatures, which would reduce EPOC. Overall, it is parsimonious that the compensatory increase in exhaustive chase duration and effort in 2°C-acclimated cunner – which was not associated with markedly greater EPOC or lactate accumulation, nor with markedly enhanced use of CrP or glycogen compared with acutely exposed 2°C fish – primarily resulted from the enhanced aerobic performance following acclimation. Improved oxygen supply could prolong exhaustive exercise by slowing CrP depletion and lactate accumulation (meaning that other factors, such as ion imbalance, may be the primary limit on exhaustive exercise in cold, dormant cunner) (Kieffer, 2010). Finally, the comparable recovery time across temperatures likely results from exercise effort and oxygen uptake responding similarly to temperature change (e.g. both were higher at warmer temperatures). Overall, our findings suggest that, even in extreme cold, EPOC in cunner is not, in and of itself, constrained; EPOC simply matches the achievable exhaustive exercise effort and associated cost of recovery, similar to other fishes (Lee et al., 2003; Svendsen et al., 2010).

### Conclusions and perspectives

Contrary to the cold performance limitation hypothesis, winter-relevant cold acclimation (2°C) in the winter-dormant cunner resulted in partial thermal compensation of multiple key metrics of whole-animal metabolic and exercise performance. Consequently, and further opposing our hypothesis, the absolute level of acclimated performance was robust in the cold, while thermal sensitivities for acclimated performance rates were similar across warm active and cold dormant temperatures, and typical for ectotherms (*Q*_10_=1–3). Compensatory thermal acclimation of performance is well known among winter-active fish species (Beddow et al., 1995; Guderley, 1990; Hvas et al., 2017; Johnston and Temple, 2002), but now appears as a conserved response among temperate fishes regardless of overwintering strategy, mirroring its surprising retention also in ‘stenothermic’ polar fish (Seebacher et al., 2005). For dormant fishes, thermal compensation could facilitate escape if uncovered by a winter-active predator. Regardless, cunner remain dormant despite strong performance following cold acclimation, strengthening our conclusion that thermal constraints on metabolic and exercise performance cannot explain the origin of dormant, and perhaps lethargic (Reeve et al., 2022), overwintering strategies among fishes. Incidentally, among ectothermic tetrapods, poor metabolic and exercise performance in the cold remains a possible explanation for winter dormancy, considering their well-recognized lethargy at low temperatures (Gregory, 1982); empirical support is lacking, though. Alternative drivers of winter dormancy in fishes should be considered, such as environmental food scarcity or the marked cold sensitivity of sensory responsiveness we found in cunner. Even if constraints underlying winter dormancy are identified, dormancy remains an innovation (sensu Miller et al., 2023), allowing certain fishes to range poleward to exploit the food-rich, warm summers while tolerating the harsh winters with minimal effort. Indeed, winter dormancy would bypass the constraint that thermal dependence of activity is thought to impose on ectotherm cold range limits (Buckley et al., 2012; Stuart-Smith et al., 2017).

Interestingly, thermal compensation in cunner only became apparent at the coldest acclimation temperature of 2°C. In other words, only at 2°C was performance increased and *Q*_10_ reduced significantly in acclimated fish relative to acutely exposed fish; this was weak or unapparent at other cold temperatures (5°C, 8°C and 11°C). Similarly, Moran et al. (2020) found that isolated muscle performance in cunner was uncompensated following 5°C acclimation (colder temperatures were not examined), and various compensatory responses underlying cold acclimation in fishes can have distinct onset temperatures (Guderley, 1990). To explain our novel observation, and building upon Guderley (1990), we propose the ‘wait-for-winter’ hypothesis: compensatory cold acclimation responses only intensify once temperature becomes low enough to (1) reliably signal the arrival of a consistent frigid winter environment and/or (2) substantially constrain performance in the absence of compensation (e.g. our 2°C acute exposures). Because acclimation can be energetically costly and temperature fluctuates widely during seasonal cooling, a ‘wait-for-winter’ strategy would optimize the cost of environment–phenotype matching by initiating plasticity only when cue reliability was high (Wilson and Franklin, 2002). ‘Wait-for-winter’ thermal plasticity could be common across temperate fishes with varied overwintering strategies, because the winter environment brings ubiquitous thermal and energetic challenges.

## Supplementary Material

10.1242/jexbio.246741_sup1 Supplementary information
