# Supplementary material for: Thermal constraints on exercise and metabolic performance do not explain the use of dormancy as an overwintering strategy in the cunner (Tautogolabrus adspersus)
Source: J Exp Biol. 2024 Jan 11;227(1):jeb246741. doi: 10.1242/jeb.246741 (PMC10906487; doi:10.1242/jeb.246741)
Supplement: Supplementary information [file jexbio-227-246741-s1.pdf]

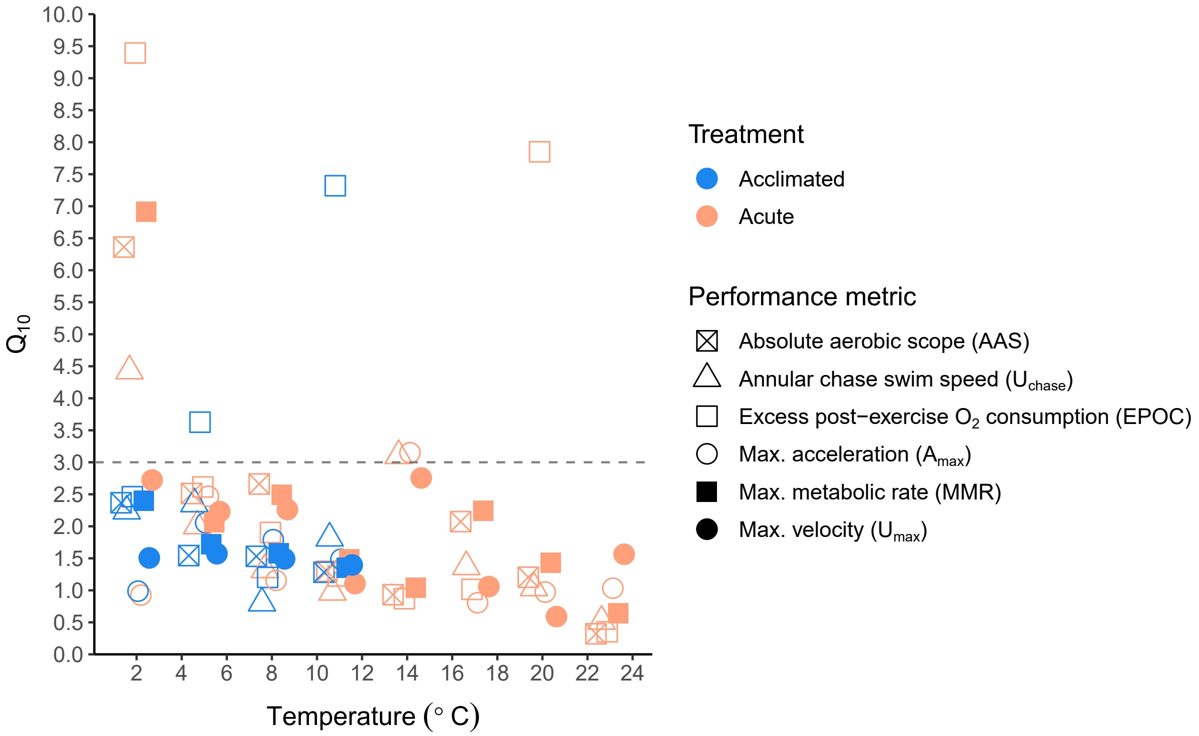

Fig. S1. Thermal sensitivity of cunner performance after acute temperature change (3°C hr^{-1} using 14°C-acclimated animals) (blue symbols) or acclimation (5-7 weeks) to 14, 11, 8, 5, or 2°C (pink symbols). The winter dormancy threshold temperature of cunner is 7-8°C (Reeve et al., 2022). Each data point represents the Q_{10} value associated with the change in the mean value of each performance metric (excluding non-rate performance, i.e., exhaustive chase duration, EPOC recovery time, responsiveness to C-start stimuli) over a 3°C interval; e.g., a data point at 2°C is the Q_{10} value from 5-2°C. Q_{10} values above the dashed grey line infer a thermal constraint, i.e., greater than the typical thermal sensitivity of metabolism and locomotor performance in fishes (Q_{10}=1-3; Seebacher et al., 2015). There are no acclimated data points >14°C because fish were not acclimated above 14°C. Different groups of cunner were used for each acute or acclimated temperature exposure, hence the use of mean performance at each temperature in calculations.

Journal of Experimental Biology • Supplementary information

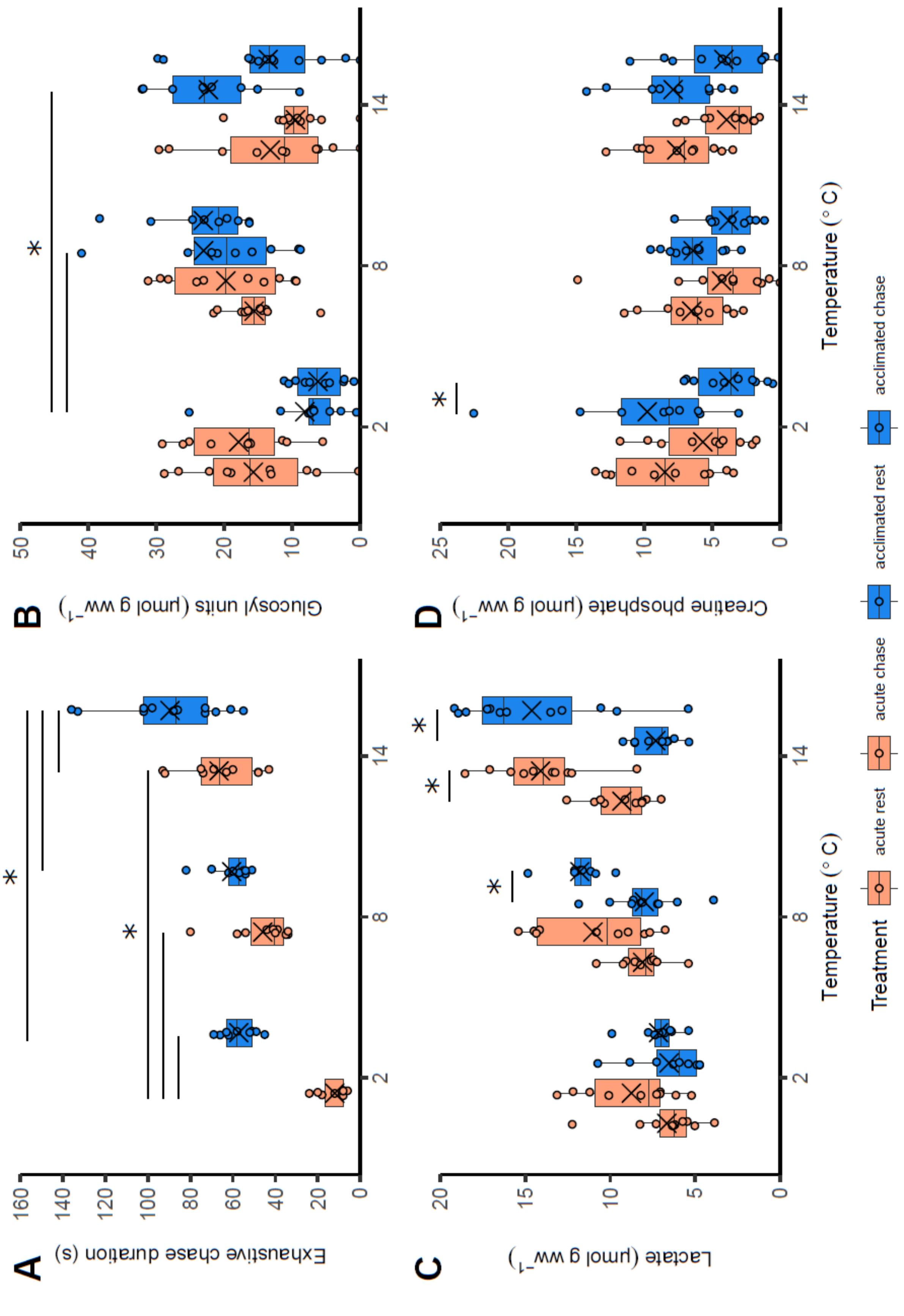
A
Exhaustive chase duration (s)
B
Glucosyl units (µmol g ww⁻¹)
C
Lactate (µmol g ww⁻¹)
D
Creatine phosphate (µmol g ww⁻¹)
Temperature (° C)
Treatment
acute rest
acute chase
acclimated rest
acclimated chase
*

Fig. S2. The effect of exhaustive chase exercise on white muscle metabolite contents in cunner acutely exposed (3°C hr^{-1} using 14°C-acclimated animals) or acclimated (7-8 weeks) to 14, 8, or 2°C (Experiment 2B). Panels show the exhaustive chase duration (A) for the chased fish and the white muscle contents of glycogen (B), lactate (C), and creatine phosphate (D) in the chased fish and resting (control) fish. Box plot elements: ×, mean; horizontal line, median; box limits, 25th and 75th percentiles; whiskers, percentiles±IQR*1.5; circles, individual data values (*n*=see Table 1). For exhaustive chase duration (panel A), * indicates a significant difference between acute and acclimated groups within the same temperature, or between temperatures within the acute or acclimated groups. For panels B, C, and D, * indicates a significant difference between rest and chase within acute or acclimated groups at each temperature, or between rest groups within acute or acclimated groups across temperatures, or between chased groups within acute or acclimated groups across temperatures (ANOVA with Tukey HSD post-hoc tests, $p<0.05$).

Journal of Experimental Biology • Supplementary information

Table S1. Body masses and total lengths of cunner used in each experiment.

		Experiment 1		Experiment 2A		Experiment 2B		
Temperature (°C)	Exposure	Mass	Total length	Mass	Total length	Resting or exercised	Mass	Total length
2	Acute	9.4 ± 0.5	9.0 ± 0.2	5.3 ± 0.5	7.3 ± 2.0	Resting	6.1 ± 1.0	7.2 ± 1.3
						Exercised	5.7 ± 0.7	7.1 ± 1.4
	Acclimated	8.7 ± 0.5	8.7 ± 0.2	4.8 ± 0.4	6.2 ± 1.4	Resting	4.1 ± 0.4	6.0 ± 1.2
						Exercised	4.6 ± 0.5	5.9 ± 1.4
5	Acute	10.5 ± 0.7	9.3 ± 0.2	6.1 ± 0.4	7.7 ± 1.7	*NA*	*NA*	*NA*
	Acclimated	9.7 ± 0.5	8.9 ± 0.2	4.7 ± 0.3	6.0 ± 1.3	*NA*	*NA*	*NA*
8	Acute	11.0 ± 0.7	9.4 ± 0.2	5.1 ± 0.4	7.7 ± 1.1	Resting	6.1 ± 0.3	7.3 ± 1.3
						Exercised	6.1 ± 0.4	7.6 ± 1.0
	Acclimated	10.6 ± 0.7	9.1 ± 0.2	5.1 ± 0.2	6.1 ± 1.5	Resting	5.4 ± 0.4	7.0 ± 1.2
						Exercised	5.0 ± 0.3	6.9 ± 1.3
11	Acute	10.3 ± 0.6	9.3 ± 0.2	5.1 ± 0.4	7.4 ± 1.5	*NA*	*NA*	*NA*
	Acclimated	11.9 ± 0.6	9.7 ± 0.2	6.2 ± 0.3	6.4 ± 1.4	*NA*	*NA*	*NA*
14	Acute	10.4 ± 0.5	9.3 ± 0.2	5.9 ± 0.4	7.4 ± 1.9	Resting	5.6 ± 0.3	6.9 ± 1.8
						Exercised	5.8 ± 0.5	6.8 ± 1.8
	Acclimated	11.1 ± 0.7	9.3 ± 0.2	6.0 ± 0.8	6.6 ± 1.6	Resting	5.7 ± 0.6	7.1 ± 1.4
						Exercised	5.8 ± 0.3	7.0 ± 1.2
17	Acute	10.4 ± 0.6	9.1 ± 0.2	6.0 ± 0.4	7.6 ± 1.6	*NA*	*NA*	*NA*
20	Acute	9.2 ± 0.7	8.7 ± 0.2	5.1 ± 0.4	7.4 ± 1.6	*NA*	*NA*	*NA*
23	Acute	9.1 ± 0.5	8.9 ± 0.2	5.4 ± 0.3	7.6 ± 1.2	*NA*	*NA*	*NA*
26	Acute	9.9 ± 0.8	9.1 ± 0.2	5.0 ± 0.3	7.8 ± 1.4	*NA*	*NA*	*NA*

Data are presented as means ± s.e.m. Body mass is presented in g and total length in cm. NA, not applicable. See supporting data file for sample sizes.

Journal of Experimental Biology • Supplementary information

Table S2. The pre-stimulation variables during the C-start test (Experiment 1) following acute cooling (3°C hr^{-1} using 14°C-acclimated cunner) or temperature acclimation (5-6 weeks). The variables are distance to the arena wall in body lengths (BL), distance to the stimulus in BL, and angle of the fish relative to the stimulus (°).

Temperature (°C)	Exposure	Sample size	Wall distance (BL)	Stimulus distance (BL)	Stimulus angle (°)
2	Acute	14	2.4 ± 0.3	0.2 ± 0.2	168.3 ± 11.7
	Acclimated	14	1.0 ± 0.3	0.5 ± 0.2	107.7 ± 23.2
5	Acute	15	0.8 ± 0.1	1.0 ± 0.4	114.9 ± 18.5
	Acclimated	14	0.8 ± 0.3	1.5 ± 0.4	33.5 ± 13.5
8	Acute	16	0.4 ± 0.1	1.0 ± 0.1	55.9 ± 15.2
	Acclimated	14	0.8 ± 0.1	1.4 ± 0.1	39.1 ± 9.0
11	Acute	16	0.7 ± 0.2	1.2 ± 0.2	50.6 ± 13.6
	Acclimated	14	0.8 ± 0.2	1.2 ± 0.2	47.1 ± 9.8
14	Acute	16	0.9 ± 0.2	1.3 ± 0.2	53.1 ± 12.9
	Acclimated	14	0.6 ± 0.1	1.2 ± 0.1	23.9 ± 7.1
17	Acute	14	0.8 ± 0.3	1.8 ± 0.3	45.5 ± 14.1
20	Acute	15	0.5 ± 0.1	1.2 ± 0.1	40.9 ± 11.8
23	Acute	10	1.4 ± 0.4	1.6 ± 0.4	62.0 ± 20.9
26	Acute	13	0.8 ± 0.4	1.3 ± 0.3	38.6 ± 13.2

Data are presented as means ± s.e.m.

Journal of Experimental Biology • Supplementary information

Table S3. Annular chase duration in seconds (s) and total distance swam in body lengths (BL) by cunner during the annular chase swimming speed test (Experiment 1) following acute cooling (3°C hr^{-1} using 14°C-acclimated animals) or temperature acclimation (5-6 weeks).

Temperature (°C)	Exposure	Sample size	Annular chase duration (s)	Distance swam (BL)
2	Acute	14	31.6 ± 3.6	100.8 ± 15.6
	Acclimated	14	49.0 ± 5.7	243.0 ± 43.0
5	Acute	15	76.9 ± 10.6	401.3 ± 70.6
	Acclimated	14	61.6 ± 6.3	375.6 ± 63.6
8	Acute	16	88.9 ± 12.8	555.6 ± 92.3
	Acclimated	14	72.5 ± 7.1	565.4 ± 82.0
11	Acute	13	142.2 ± 13.8	956.1 ± 109.3
	Acclimated	14	74.2 ± 5.2	530.8 ± 55.6
14	Acute	15	153.8 ± 26.1	1105.9 ± 234.4
	Acclimated	14	99.9 ± 5.8	840.7 ± 64.7
17	Acute	14	77.3 ± 9.5	765.0 ± 116.4
20	Acute	15	71.3 ± 4.5	745.1 ± 52.1
23	Acute	10	44.1 ± 6.3	473.0 ± 81.8
26	Acute	13	32.5 ± 5.1	288.4 ± 52.6

Data are presented as means ± s.e.m.

Journal of Experimental Biology • Supplementary information

Table S4. The Q_{10} and percent change values for performance metrics (and standard metabolic rate, a cost of basic maintenance) in cunner following acute temperature change (3°C hr^{-1} using 14°C-acclimated animals) or temperature acclimation (5-7 weeks) above and below the winter dormancy threshold temperature (7-8°C for cunner (Reeve et al., 2022)). Specifically, the Q_{10} and percent change values were calculated over 6°C intervals above and below the winter dormancy threshold temperature as well as following acute warming of 14°C-acclimated animals to 20°C (i.e., 14-20°C, 14-8°C: normal activity; 8-2°C: dormant). Different groups of cunner were used for each acute or acclimated temperature exposure, so each Q_{10} and percent change value was calculated using the mean performance of each group (hence, the lack of error estimates). Q_{10} values are not provided for non-rate performance metrics (i.e., exhaustive chase duration, EPOC recovery time, and responsiveness to C-start stimuli).

Performance metric	Exposure	Temperature interval (°C)	Q_{10}	Percent change (%)
U_{max}	Acute	14-20	1.7	37.9
		14-8	1.6	-24.0
		8-2	2.5	-41.8
	Acclimated	14-8	1.4	-19.8
		8-2	1.5	-22.8
A_{max}	Acute	14-20	1.6	32.2
		14-8	1.3	-12.8
		8-2	1.5	-22.0
	Acclimated	14-8	1.6	-25.4
		8-2	1.4	-19.1
Responsiveness to C-start stimulus	Acute	14-20	NA	9.9
		14-8	NA	0.0
		8-2	NA	-86.4
	Acclimated	14-8	NA	-16.1
		8-2	NA	-67.3
U_{chase}	Acute	14-20	2.1	54.3
		14-8	1.1	-7.2
		8-2	3.0	-48.1
	Acclimated	14-8	1.3	-10.6
		8-2	2.3	-39.2
Exhaustive chase duration	Acute	14-20	NA	-26.6
		14-8	NA	-30.1
		8-2	NA	-58.3
	Acclimated	14-8	NA	-25.5
		8-2	NA	-5.0

Journal of Experimental Biology • Supplementary information

SMR	Acute	14-20	2.4	69.9
		14-8	2.3	-40.1
		8-2	2.6	-44.7
	Acclimated	14-8	1.8	-30.5
		8-2	3.1	-49.1
MMR	Acute	14-20	1.5	29.0
		14-8	1.9	-32.7
		8-2	3.8	-55.4
	Acclimated	14-8	1.5	-20.6
		8-2	2.0	-33.9
Absolute aerobic scope	Acute	14-20	1.4	22.0
		14-8	1.9	-31.5
		8-2	4.0	-57.0
	Acclimated	14-8	1.4	-18.8
		8-2	1.9	-31.5
Total EPOC	Acute	14-20	0.9	-3.6
		14-8	1.5	-22.8
		8-2	4.9	-61.6
	Acclimated	14-8	2.9	-48.6
		8-2	3.0	-47.6
EPOC recovery time	Acute	14-20	NA	-1.5
		14-8	NA	54.2
		8-2	NA	-33.2
	Acclimated	14-8	NA	31.1
		8-2	NA	-47.4

U_{max}, maximum burst velocity; A_{max}, maximum burst acceleration; U_{chase}, annular chase swim speed; SMR, standard metabolic rate; MMR, maximum metabolic rate; EPOC, excess post-exercise oxygen consumption. NA, not applicable.

Journal of Experimental Biology • Supplementary information
